# The Value of Genetic Information for Diabetes Risk Prediction – Differences According to Sex, Age, Family History and Obesity

**DOI:** 10.1371/journal.pone.0064307

**Published:** 2013-05-20

**Authors:** Kristin Mühlenbruch, Charlotte Jeppesen, Hans-Georg Joost, Heiner Boeing, Matthias B. Schulze

**Affiliations:** 1 Department of Molecular Epidemiology, German Institute of Human Nutrition Potsdam-Rehbruecke, Nuthetal, Germany; 2 Department of Pharmacology, German Institute of Human Nutrition Potsdam-Rehbruecke, Nuthetal, Germany; 3 Department of Epidemiology, German Institute of Human Nutrition Potsdam-Rehbruecke, Nuthetal, Germany; 4 German Center for Diabetes Research, Neuherberg, Germany; German Institute of Human Nutrition Potsdam-Rehbruecke, Germany

## Abstract

**Background:**

Genome-wide association studies have identified numerous single nucleotide polymorphisms associated with type 2 diabetes through the past years. In previous studies, the usefulness of these genetic markers for prediction of diabetes was found to be limited. However, differences may exist between substrata of the population according to the presence of major diabetes risk factors. This study aimed to investigate the added predictive value of genetic information (42 single nucleotide polymorphisms) in subgroups of sex, age, family history of diabetes, and obesity.

**Methods:**

A case-cohort study (random subcohort *N* = 1,968; incident cases: *N* = 578) within the European Prospective Investigation into Cancer and Nutrition Potsdam study was used. Prediction models without and with genetic information were evaluated in terms of the area under the receiver operating characteristic curve and the integrated discrimination improvement. Stratified analyses included subgroups of sex, age (<50 or ≥50 years), family history (positive if either father or mother or a sibling has/had diabetes), and obesity (BMI< or ≥30 kg/m^2^).

**Results:**

A genetic risk score did not improve prediction above classic and metabolic markers, but – compared to a non-invasive prediction model – genetic information slightly improved the area under the receiver operating characteristic curve (difference [95%-CI]: 0.007 [0.002–0.011]). Stratified analyses showed stronger improvement in the older age group (0.010 [0.002–0.018]), the group with a positive family history (0.012 [0.000–0.023]) and among obese participants (0.015 [−0.005–0.034]) compared to the younger participants (0.005 [−0.004–0.014]), participants with a negative family history (0.003 [−0.001–0.008]) and non-obese (0.007 [0.000–0.014]), respectively. No difference was found between men and women.

**Conclusion:**

There was no incremental value of genetic information compared to standard non-invasive and metabolic markers. Our study suggests that inclusion of genetic variants in diabetes risk prediction might be useful for subgroups with already manifest risk factors such as older age, a positive family history and obesity.

## Introduction

According to the most recent meta-analysis of genome-wide association studies, 63 individual SNPs have now been linked with diabetes risk [1]. However, these variants explain only ∼5.7% of variance in disease susceptibility [1]. Genetic markers have also been frequently compared with established risk factors for type 2 diabetes in terms of their usefulness for predicting risk [2]. For example, we have previously reported that information on 20 SNPs is not informative for predicting future diabetes in the EPIC-Potsdam study [3]. Overall, prospective studies showed limited predictive value of genetic markers in general, and particularly if compared to classical non-genetic risk factors [4]. However, few studies indicate that prediction by genetic variants might be informative among specific subgroups, e.g. individuals who are younger (<50 years) [5], [6], or who are obese [6]. However, a systematic comparison of genetic and non-genetic risk factors in subgroups of a prospective study that allows an accurate determination of the diabetes risk is still lacking. Our aim was therefore to evaluate if the predictive value of a large set of genetic variants differed between subgroups according to sex, age, family history, and BMI.

## Methods

### Ethics statement

The ethics committee of the State of Brandenburg, Germany, gave approval for the study and written informed consent was obtained from all participants.

### Study design and participants

The European Prospective Investigation into Cancer and Nutrition (EPIC)–Potsdam study is a population-based cohort study of 27,548 participants recruited from Potsdam, Germany, in the years 1994–1998 [7]. Participants were mainly aged 35–65 years at baseline. Follow-up assessment was performed every 2 to 3 years to identify incident type 2 diabetes cases. Over a mean follow-up time of 7 years, 849 incident cases were identified. The diagnosis of incident cases was based on self-reports in a questionnaire and verification by physicians.

We used a case-cohort nested within the prospective EPIC-Potsdam cohort for evaluation of genetic risk factors. Out of 26,444 study participants with blood samples collected at baseline a random subcohort with 2,500 participants was selected. This subcohort is representative for the full cohort and baseline characteristics showed no significant differences [8].Participants with prevalent type 2 diabetes at baseline, abnormal baseline plasma glucose levels or more than 9 missing data on SNPs were excluded from analysis leaving 1,968 participants in the subcohort. Of the 801 incident cases identified during follow-up in the cohort with blood samples, 578 cases remained after similar exclusions.

Baseline information was used to calculate the German Diabetes Risk Score (GDRS), a validated prediction equation developed in the EPIC-Potsdam cohort including the following non-invasive measures: age (years), height (cm), waist circumference (cm), prevalent hypertension (yes vs. no), physical activity (h/week), smoking (currently smoking≥20 cig./d, ex-smoking vs. never smoker or currently smoking <20 cig./d), alcohol intake (moderate consumption [10–40 g/d] vs. low or high consumption), intake of red meat (150 g/d), intake of wholegrain bread (50 g/d) and coffee consumption (150 ml cup/d). [9]. Diet was assessed with a validated semiquantitative food frequency questionnaires (FFQ) including 148 food items. Frequencies were measured in 10 categories and portion sizes were estimated from photographs of standard portion sizes [10], [11], [12]. Information on family history of diabetes was obtained from self-reports in a questionnaire, and body mass index (BMI) was calculated from measures of height and weight (kg/m^2^) in a physical examination. Measurements of metabolic markers were described in previous reports [3], [7].

### Genotyping

Genotyping of 20 previously analyzed SNPs associated with type 2 diabetes was performed with Taqman technology (Applied Biosystems, Foster City, CA) [3]. For the current analyses, 23 additional SNPs were genotyped by KBioscience (http://www.kbioscience.co.uk) using KASP SNP genotyping system. This is a competitive allele-specific PCR incorporating a FRET quencher cassette. The overall set of SNPs was selected to reflect common diabetes-associated single nucleotide polymorphisms and to be largely identical to the set of SNPs evaluated in sub-group analysis the Framingham Offspring study [5] for better comparability. The accuracy of genotyping was independently assessed to be between 0 and 0.2%, with reproducibility at 99.9% and success rate of 96.5%. For single SNPs frequency of missing genotype information was lower than 6.5%. All SNPs were in Hardy-Weinberg equilibrium (*p*>0.001), except for rs5945326 (near DUSP9 gene) and hence this SNP was excluded from analysis.

### Statistical analyses

We assumed an additive model for each SNP with values of 0, 1 and 2 for the number of risk alleles and analyzed the predictive value of the SNPs using a count genetic score. For participants with missing genotypes the genetic score was standardized to score values for participants with complete genotype information [13].

The incremental value of metabolic markers and the count genetic score was investigated with several different prediction models. The discriminatory ability of each model was determined with the areas under receiver operating characteristic curves (ROC-AUCs) using logistic regression analysis. Model comparisons of a sparser with an extended model were used to assess the improvement in prediction with difference in ROC-AUCs and 95% confidence intervals (95% CI) calculated with the Delong test [14]. The integrated discrimination improvement (IDI) was calculated with predicted risks from logistic regression [15]. Model comparisons were repeated in subgroups of sex, age (<50, ≥50 years of age), family history of diabetes (positive: father, mother or at least one sibling has type 2 diabetes) and obesity (positive: BMI≥30 kg/m^2^).

All statistical analyses were performed with SAS (Version 9.2, Enterprise Guide 4.3, SAS Institute Inc., Cary, NC, USA). The significance level was defined with a two-tailed *p*-value of <0.05.

## Results

Baseline characteristics of the random subcohort of EPIC-Potsdam and incident cases are presented in table 1. Incident cases were, compared to the subcohort, more likely to be males, were on average older, had a higher BMI, and had a wider waist circumference. Proportions of hypertensive participants and former or current smokers were larger among incident cases. Diabetes risk quantified with the GDRS was considerably higher among incident cases compared to the subcohort participants. Also, concentrations of biochemical markers reflected a higher baseline risk for incident cases compared to the subcohort. Regarding the genetic score, incident cases had slightly higher number of risk alleles than the members in the subcohort.

**Table 1:** Baseline characteristics for the case-cohort of EPIC-Potsdam.

Characteristic	Random subcohort	Incident cases of Type 2 diabetes
No. of participants	1,968	578
Sex (% male)	37.0	58.8
Age (years)	49.4±9.0	54.6±7.5
BMI (kg/m^2^)	25.9±4.2	30.4±4.5
Waist circumference (cm)	85.2±12.6	100.1±12.2
Body height (cm)	167.6±8.5	168.6±8.7
Prevalent hypertension (%)	30.5	58.0
Moderate alcohol consumption (% 10–40 g/d)	33.8	30.8
Former smoker (%)	31.4	44.3
Current smoker (%≥20 units/d)	5.7	9.2
Intake of wholegrain bread (50 g portion/d)	0.9±1.1	0.8±0.9
Coffee consumption (150 ml cup/d)	2.8±2.1	2.7±2.1
Intake of red meat (150 g portion/d)	0.3±0.2	0.3±0.2
Physical activity (h/week)	6.2±5.9	6.0±5.8
Score points GDRS	441.1±120.9	595.0±99.4
*Biochemical markers*		
HbA1c (%)	6.42 (6.40–6.45)	7.13 (7.06–7.19)
Random glucose (mg/dl)	95.0 (94.3–95.8)	110.1 (108.3–112.0)
Adiponectin (µg/ml)	7.25 (7.10–7.41)	5.10 (4.91–5.30)
HDL-cholesterol (mg/dl)	51.4 (50.7–51.9)	42.8 (41.9–43.6)
Triglycerides (mg/dl)	104.7 (102.1–107.4)	166.2 (158.8–173.9)
hs-CRP (mg/l)	0.72 (0.68–0.77)	1.73 (1.56–1.92)
γ-glutamyltransferase (U/l)	17.9 (17.3–18.6)	33.4 (31.3–35.6)
alanine-aminotransferase (U/l)	19.4 (19.0–19.9)	29.1 (27.9–30.5)
Genetic risk score (20 SNPs)	19.2±2.9	19.8±2.9
Genetic risk score (42 SNPs)	42.6±4.2	43.7±4.0

Presented values are means ± standard deviation (SD) and for biochemical markers geometric means are presented with 95% confidence intervals (95% CI).

The genetic loci and risk-allele frequencies are listed in Table S1. Risk-allele frequencies ranged from 0.09 to 0.94 in the random EPIC-subcohort and were comparable with prior reports on allele frequencies [5], [16], [17] or HapMap-CEU and 1000 Genomes data [18].

Table 2 shows comparisons of models without or with inclusion of the count genetic score. Discrimination for a model including only the 42 SNP genetic score was weak (ROC-AUC: 0.579; 95% CI: 0.552–0.605). However, when adding the genetic score to the GDRS ROC-AUC increased from 0.846 to 0.853 (delta: 0.007 [95% CI: 0.002–0.011]). Additionally including genetic markers to a model containing the GDRS, glucose, A1C, triglycerides, HDL cholesterol, γ-glutamyltransferase and alanine aminotransferase showed small differences in ROC-AUC (0.002 [−0.001–0.004]) but without significance.

**Table 2:** Improvement in Discrimination by Adding Genetic Information to the GDRS and Metabolic Biomarkers in the Case-Cohort of EPIC-Potsdam.

	ROC		IDI	
	C statistic (95% CI)	delta ROC-AUC (95% CI)	Absolute IDI (95% CI)	Relative IDI (%)
Genetic markers (42 SNPs) only	0.579 (0.552–0.605)	-	-	-
GDRS only	0.846 (0.829–0.863)	Ref.	Ref.	Ref.
GDRS and genetic markers (42 SNPs)	0.853 (0.836–0.869)	0.007 (0.002 – 0.011)	0.0154 (0.0152–0.0157)	5.44
GDRS, glucose, A1C, triglycerides, HDL cholesterol, γ-glutamyltransferase, and alanine aminotransferase	0.899 (0.885–0.913)	Ref.	Ref.	Ref.
GDRS, glucose, A1C, triglycerides, HDL cholesterol, γ-glutamyltransferase, alanine aminotransferase and genetic markers (42 SNPs)	0.901 (0.887–0.914)	0.002 (−0.001–0.004)	0.0071 (0.0069–0.0072)	1.66

Stratified analyses for prediction models including the count genetic score, GDRS or count genetic score along with the GDRS are presented in table 3. The discriminative ability of the genetic score alone was weak in both men and women. Also, the predictive value of the genetic score added to the GDRS was similar in men and women (differences in ROC-AUC: 0.006 and 0.008, IDI: 6.20 and 6.24%, respectively). Analyses stratified by age showed lower ROC-AUCs for the GDRS and the genetic score in the upper age group. When the genetic score was included along with the GDRS, improvement was more apparent in the older age group (difference of ROC-AUC: 0.010, IDI: 7.50%) compared to the younger group (0.005, 5.25%). We observed a slightly higher ROC-AUC for the genetic risk score among participants with a positive family history, while the GDRS discriminated slightly better in the group with a negative history. Improvement by genetic information was larger among participants with a positive family history (delta ROC-AUC: 0.012, relative IDI: 8.71%) compared to participants with a negative family history. With regard to BMI subgroups, both the GDRS and genetic score showed a better discrimination in the group without obesity. Although non-significant, improvement in discrimination was larger in the obese group.

**Table 3:** Stratified Analyses of the GDRS and GRS with 42 SNPs for Sex, Age, Family History of Diabetes, and Obesity Status.

	ROC		IDI	
	C statistic (95% CI)	delta ROC-AUC (95% CI)	Absolute IDI (95% CI)	Relative IDI (%)
***Women*** * (238 cases and 1,216 non-cases)*				
Genetic markers (42 SNPs) only	0.581 (0.542–0.621)	-	-	-
GDRS only	0.855 (0.832–0.879)	Ref.	Ref.	Ref.
GDRS and genetic markers (42 SNPs)	0.861 (0.838–0.884)	0.006 (−0.00–0.013)	0.0158 (0.0155–0.0162)	6.20
***Men*** * (340 cases and 701 non-cases)*				
Genetic markers (42 SNPs) only	0.582 (0.546–0.619)	-	-	-
GDRS only	0.812 (0.785–0.839)	Ref.	Ref.	Ref.
GDRS and genetic markers (42 SNPs)	0.820 (0.793–0.846)	0.008 (−0.000–0.016)	0.0161 (0.0158–0.0165)	6.24
***Age<50 years*** * (154 cases and 996 non-cases)*				
Genetic markers (42 SNPs) only	0.589 (0.540–0.637)	-	-	-
GDRS only	0.883 (0.855–0.910)	Ref.	Ref.	Ref.
GDRS and genetic markers (42 SNPs)	0.887 (0.861–0.914)	0.005 (−0.004–0.014)	0.0169 (0.0164–0.0173)	5.25
***Age≥50 years*** * (424 cases and 921 non-cases)*				
Genetic markers (42 SNPs) only	0.577 (0.545–0.609)	-	-	-
GDRS only	0.788 (0.763–0.813)	Ref.	Ref.	Ref.
GDRS and genetic markers (42 SNPs)	0.798 (0.774–0.822)	0.010 (0.002–0.018)	0.0161 (0.0159–0.0164)	7.50

	ROC		IDI	
	C statistic (95% CI)	delta ROC-AUC (95% CI)	Absolute IDI (95% CI)	Relative IDI (%)
***Negative Family History of Diabetes*** * (295 cases and 1,272 non-cases)*				
Genetic markers (42 SNPs) only	0.566 (0.530–0.602)	-	-	-
GDRS only	0.851 (0.830–0.872)	Ref.	Ref.	Ref.
GDRS and genetic markers (42 SNPs)	0.855 (0.833–0.876)	0.003 (−0.001–0.008)	0.0098 (0.0095–0.0100)	3.58
***Positive Family History*** a ***of Diabetes*** * (202 cases and 456 non-cases)*				
Genetic markers (42 SNPs) only	0.599 (0.551–0.647)	-	-	-
GDRS only	0.841 (0.809–0.872)	Ref.	Ref.	Ref.
GDRS and genetic markers (42 SNPs)	0.852 (0.822–0.883)	0.012 (0.000–0.023)	0.0266 (0.0261–0.0271)	8.71
***Non-obese*** * (301 cases and 1,650 non-cases)*				
Genetic markers (42 SNPs) only	0.599 (0.565–0.634)	-	-	-
GDRS only	0.832 (0.809–0.855)	Ref.	Ref.	Ref.
GDRS and genetic markers (42 SNPs)	0.839 (0.817–0.861)	0.007 (0.000–0.014)	0.0164 (0.0161–0.0167)	7.55
***Obese*** b *(277 cases and 267 non-cases)*				
Genetic markers (42 SNPs) only	0.572 (0.524–0.620)	-	-	-
GDRS only	0.705 (0.662–0.749)	Ref.	Ref.	Ref.
GDRS and genetic markers (42 SNPs)	0.720 (0.677–0.763)	0.015 (−0.005–0.034)	0.0202 (0.0198–0.0206)	15.87

a A positive family history of diabetes was defined as either the mother or the father or a sibling suffered from type 2 diabetes.

b Obesity was defined as BMI≥30 kg.

## Discussion

We observed that prediction based on a large number of single nucleotide polymorphisms is not accurate, regardless of subgroups with different risk according to sex, age, family history, or obesity. Prediction based on a model with non-invasive risk factors was slightly improved by genetic information, but not if established biochemical risk markers were also considered. We also observed that improvement in prediction by genetic information beyond classical risk factors was slightly larger among older or obese participants and participants with a family history of diabetes.

Genetic markers alone showed a discriminatory ability (ROC-AUC) between 0.54 [19] and 0.68 [20] in previous studies. Our results, based on a genetic risk score including 42 SNPs, are comparable with these findings. Although we used a larger set of SNPs compared to most previous studies, acceptable discrimination by genetic information alone will require identification of many more common variants (usually with small effects) or rare variants with stronger effects [21].

We have previously reported that metabolic markers improved discrimination of the German diabetes risk score substantially, whereas a genetic risk score including 20 SNPs did not [3]. Our current analyses showed comparable results with no added value of 42 SNPs beyond metabolic markers. These results are in accordance with previous studies [19], [22] suggesting that prediction models involving lifestyle and biochemical predictors and showing very good discrimination are not improved by the known genetic markers [23]. Still, we observed an improvement in discrimination with the genetic risk score if only a non-invasive model served as the reference. Thus, genetic profiling could be an alternative to the determination of biochemical markers. However, the improvement by genetic information is so far much smaller than that observed with conventional biochemical risk markers, such as plasma glucose, HbA1c, or plasma lipids.

Previous reports suggest that genetic risk prediction might be more useful in younger populations [5], [24], [25]. In the Framingham Offspring study Meigs et al. found a substantially better model improvement by a genotype score in participants being <50 years of age compared to older [25]. The same trend was observed by de Miguel-Yanes et al. when evaluating an extended set of SNPs (40) in the same study population [5]. Data from the Malmö and Botnia studies support this notion: the ability of genetic risk factors to predict future type 2 diabetes improved with an increasing duration of follow-up, in contrast to lifestyle-related risk factors [24]. However, a recent study by Vassy et al. among young adults did not see an improvement in prediction by genetic information over routine clinical measurements [26]. Similarly, our results did not support the hypothesis that prediction by genetic information is more accurate in younger individuals. To the contrary, we found that 42 SNPs, an almost identical set compared to de Miguel-Yanes et al. [5], improved prediction more among older participants (≥50 years) than among younger. Although we cannot rule out that the larger improvement among older participants in our study could be due to the relatively lower discrimination achieved by the lifestyle-related prediction model, the Framingham Offspring study observed larger improvements among younger participants despite the fact that the baseline model without genetic risk factors actually showed slightly better discrimination as well compared to older participants. While these different results might be explained by differences in the study populations [27], in the study design and in the identification of diabetes cases, or in the baseline risk factor models considered, our results support that genetic risk might affect people with an adverse risk profile (e.g. older age) more likely than with a healthy risk profile [4]. This is further supported by our observation that improvement in discrimination by genetic information was larger among participants who had a family history of diabetes or who were obese.

To our knowledge, no previous study reported stratified analysis by family history of diabetes. Some studies suggest that the strength of the association between genetic risk scores and diabetes depends on family history, but this is only indirect support for our observation [13], [20]. Talmud et al. hypothesized that the inclusion of family history in a reference model could weaken the added predictive value of genetic risk markers, if it was part of the family history complex [19]. However, recent results from the InterAct consortium suggest that the currently known diabetes gene variants only explain a very minor proportion of excess risk associated with family history [28].

We found that the discriminatory ability of the 42 SNPs alone was slightly better in the non-obese group compared to the obese, however, discrimination was generally poor irrespective of obesity status. Van Hoek et al. showed similar results for low compared to high BMI groups (cut-off 26 kg/m^2^). However, genetic information resulted in a stronger improvement in ROC-AUC among obese compared to non-obese participants in our study. This difference could be due to the relatively lower discrimination among obese participants achieved by the lifestyle-related prediction model. However, the improvement in ROC-AUC reached not statistical significance in the obese group, but this might mainly reflect the smaller sample size particularly for non-cases. No other study investigated improvement of prediction models by genetic markers in subgroups of BMI. While two studies observed stronger associations of genetic risk scores with type 2 diabetes risk in obese people compared to non-obese after adjustment for age and sex [13], [20], the extent to which genetic information improve prediction has not been investigated.

Several limitations of our study need to be considered. While a strength of our study is its prospective design, we included in our analysis only clinical cases of diabetes identified by self-reports and did not screen our study population for unknown diabetes during follow-up. Thus, our results may not be generalizable to patients who are identifiable only by screening. Further, we cannot rule out that diabetes cases include subtypes such as latent autoimmune diabetes of adults (LADA) which might have affected our results. Also, the prospective design rendered it necessary to exclude all prevalent diabetes cases at baseline. Thus, our results reflect genetic prediction in middle-aged individuals but not prediction at birth. However, a prospective design is more meaningful than a case-control design if prediction by genetic variants is compared with prediction by anthropometric and lifestyle-related risk factors. We based our analysis on a large number of established diabetes SNPs, however, we cannot rule out that a more comprehensive list of SNPs would be more useful for prediction purposes, although this appears to be unlikely [4]. We have evaluated the discriminatory predictive power of genetic markers using ROC analyses and reclassification (IDI). It has been suggested that evaluation of different risk prediction models should also include the net reclassification improvement [15], [29]. However, we have recently reported that the absence of established risk classes for diabetes introduces large subjectivity to such analyses [30]. Similar to previous studies [5], [6], the comparison of delta ROC-AUCs between subgroups did not rely on a statistical test which might introduce subjectivity to the interpretation of the results. Our stratified analyses were based on the original GDRS with published points, but the predictive value of age or waist circumference might be different in strata of age or BMI, respectively. However, refitting the prediction models in different strata only slightly affected the ROC-AUCs and improvement stayed almost the same, so that the overall conclusion would not be changed.

In conclusion, genetic risk prediction with 42 SNPs alone was not accurate enough to be used for identification of individuals at high risk. In addition to conventional non-invasive risk factors genetic risk prediction might be used to achieve a slightly higher accuracy, however, it failed to significantly improve risk prediction with established biochemical risk factors. Although differences were not substantial, our data suggest, that genetic variants might be more useful for prediction within subgroups with already manifest risk factors, such as higher age, obesity, and a positive family history of diabetes.

## Supporting Information

Table S1 **Genetic loci and genotype frequencies among members of the EPIC-Potsdam subcohort for 42 SNPs associated with Type 2 Diabetes.**
(DOCX)
