# Correction: The Value of Genetic Information for Diabetes Risk Prediction – Differences According to Sex, Age, Family History and Obesity

**DOI:** 10.1371/annotation/65bd3a11-b821-4f10-88d2-29b69a730f21

**Published:** 2013-09-17

**Authors:** Kristin Mühlenbruch, Charlotte Jeppesen, Hans-Georg Joost, Heiner Boeing, Matthias B. Schulze

The Editor's affiliation is incorrect. The correct affiliation is: German Diabetes Center, Leibniz Center for Diabetes Research at Heinrich Heine University Duesseldorf, Germany. 

