# Supplementary material for: The Value of Genetic Information for Diabetes Risk Prediction – Differences According to Sex, Age, Family History and Obesity
Source: PLoS One. 2013 May 20;8(5):e64307. doi: 10.1371/journal.pone.0064307 (PMC3658960; doi:10.1371/journal.pone.0064307)
Supplement: Table S1 — Genetic loci and genotype frequencies among members of the EPIC-Potsdam subcohort for 42 SNPs associated with Type 2 Diabetes. (DOCX) [file pone.0064307.s001.docx]

Table S1: Genetic loci and genotype frequencies among members of the EPIC-Potsdam subcohort for 42 SNPs associated with Type 2 Diabetes

| **SNP** | **Nearby Gene** | **Percent Missing in case-cohort, EPIC-Potsdam (N=2,582)^a^** | **Risk-Allele** | **Risk-Allele Frequency in Subcohort, EPIC- Potsdam (N=1,968)** |
| --- | --- | --- | --- | --- |
| rs4607103 | ADAMTS9 | 5.38 | C | 0.75 |
| rs11708067 | ADCY5 | 3.80 | T | 0.78 |
| rs243021 | BCL11A | 3.41 | T | 0.46 |
| rs10490072 | BCL11A | 4.88 | T | 0.72 |
| rs12779790 | CDC123/CAMK1D | 3.95 | G | 0.15 |
| rs7754840 | CDKAL1 | 1.51 | C | 0.35 |
| rs10811661 | CDKN2A | 2.13 | C | 0.83 |
| rs1552224 | CENTD2 | 3.21 | T | 0.83 |
| rs13292136 | CHCHD9 | 2.48 | C | 0.94 |
| rs1153188 | DCD | 6.43 | A | 0.27 |
| rs2191349 | DGKB/TME195 | 4.53 | T | 0.55 |
| rs8050136 | FTO | 1.94 | A | 0.42 |
| rs4607517 | GCK | 3.06 | A | 0.15 |
| rs780094 | GCKR | 3.68 | G | 0.59 |
| rs10423928 | GIPR | 3.64 | A | 0.24 |
| rs2334499 | HCCA2 | 3.45 | T | 0.41 |
| rs1111875 | HHEX | 1.39 | G | 0.59 |
| rs1531343 | HMGA2 | 3.18 | C | 0.09 |
| rs7957197 | HNF1A | 3.52 | T | 0.81 |
| rs4402960 | IGF2BP2 | 2.56 | C | 0.29 |
| rs7578326 | IRS1 | 3.41 | A | 0.63 |
| rs864745 | JAZF1 | 4.88 | T | 0.50 |
| rs5219 | KCNJ11/E23K | 1.78 | T | 0.37 |
| rs231362 | KCNQ1 | 4.49 | C | 0.49 |
| rs2237892 | KCNQ1 | 2.83 | C | 0.93 |
| rs972283 | KLF14 | 3.06 | G | 0.53 |
| rs1387153 | MTNR1B | 3.33 | T | 0.29 |
| rs10923931 | NOTCH2 | 1.43 | T | 0.11 |
| rs1801282 | PPARg | 3.52 | C | 0.84 |
| rs8042680 | PRC1 | 3.14 | A | 0.10 |
| rs340874 | PROX1 | 4.26 | C | 0.53 |
| rs7593730 | RBMS1 | 3.68 | C | 0.82 |
| rs13266634 | SLC30A8 | 1.98 | C | 0.69 |
| rs4430796 | TCF2 | 4.57 | G | 0.47 |
| rs7903146 | TCF7L2 | 1.39 | T | 0.25 |
| rs7578597 | THADA | 4.38 | T | 0.88 |
| rs896854 | TP53INP1 | 4.14 | A | 0.51 |
| rs7961581 | TSPAN8/LGR5 | 4.53 | C | 0.27 |
| rs9472138 | VEGFA | 4.73 | T | 0.27 |
| rs10010131 | WFS1 | 4.45 | G | 0.59 |
| rs4457053 | ZBED3 | 3.64 | G | 0.30 |
| rs11634397 | ZFAND6 | 3.99 | G | 0.67 |

^a^ The amount of missing values due to genotyping error was calculated before exclusion of more than 9 missing SNP values which was applied for analyses.
